# Selection of chemically defined media for CHO cell fed-batch culture processes

**DOI:** 10.1007/s10616-016-0036-5

**Published:** 2016-11-29

**Authors:** Xiao Pan, Mathieu Streefland, Ciska Dalm, René H. Wijffels, Dirk E. Martens

**Affiliations:** 1 Bioprocess Engineering, Wageningen University, PO Box 16, 6700 AA Wageningen, The Netherlands; 2 Synthon Biopharmaceuticals BV, Upstream Process Development, PO Box 7071, 6503 GN Nijmegen, The Netherlands; 3 Faculty of Biosciences and Aquaculture, Nord University, 8049 Bodø, Norway

**Keywords:** Chinese hamster ovary (CHO) cell, Cell culture medium, Fed-batch, Antibody production, Metabolite profile, Phase transition

## Abstract

Two CHO cell clones derived from the same parental CHO^BC®^ cell line and producing the same monoclonal antibody (BC-G, a low producing clone; BC-P, a high producing clone) were tested in four basal media in all possible combinations with three feeds (=12 conditions) in fed-batch cultures. Higher amino acid feeding did not always lead to higher mAb production. The two clones showed differences in cell physiology, metabolism and optimal medium-feed combinations. During the phase transitions of all cultures, cell metabolism showed a shift represented by lower specific consumption and production rates, except for the specific glucose consumption rate in cultures fed by Actifeed A/B. The BC-P clone fed by Actifeed A/B showed a threefold cell volume increase and an increase of the specific consumption rate of glucose in the stationary phase. Since feeding was based on glucose this resulted in accumulation of amino acids for this feed, while this did not occur for the poorer feed (EFA/B). The same feed also led to an increase of cell size for the BC-G clone, but to a lesser extent.

**Electronic supplementary material:**

The online version of this article (doi:10.1007/s10616-016-0036-5) contains supplementary material, which is available to authorized users.

## Introduction

Chinese Hamster Ovary (CHO) cells are the main production host for recombinant therapeutic proteins (Wurm 2004). Fed-batch culture is currently the most common industrial process for CHO cell culture. For a fed-batch process volumetric productivity and product titer are important outputs. These outputs are a function of viable cell density (VCD), specific productivity (q_p_), and culture longevity, which in turn are influenced by the medium and feed composition. Selecting a suitable culture medium and feed platform is therefore pivotal for developing a fed-batch manufacturing process. Chemically defined basal media and feeds are currently the standard for CHO cell fed-batch cultures. In a fed-batch culture, CHO cells are inoculated and start to grow in a basal medium. A feed is added to the culture when a certain culture state is reached, such as when a certain cell density is reached, a certain culture time point is reached, or a certain nutrient concentration is reached. The basal medium and especially the feed should contain a balanced set of essential nutrients in a ratio that meets the demand of the cells for cell proliferation and production of the pharmaceutical protein. A feed is generally more concentrated than a basal medium to maximize culture volumetric productivity and product titer. Studies have shown that the composition of culture media and feeds can influence cell growth, protein productivity (Rodrigues et al. 2012), gene expression (Zagari et al. 2013), product quality (Costa et al. 2013; Hossler et al. 2009; Reinhart et al. 2015), and metabolism of lactate and ammonia (Zagari et al. 2013; Kyriakopoulos and Kontoravdi 2014; Luo et al. 2012; Ma et al. 2009).

To design a chemically defined medium in-house is time and labor consuming. Currently, process development of CHO cell culture usually starts with commercially available culture media systems as a basis (Rodrigues et al. 2012). Several well performing chemically defined media have become commercially available for CHO cells. These media are designed based on different strategies to reach high volumetric productivities and product titers. For instance, some media are designed to boost cell growth to a high cell density, others are designed to extend the longevity of cells or to enhance cell specific productivity. For different medium-feed combinations the richness and composition of the basal media and feeds differ widely. The optimal composition of a basal medium or a feed is highly dependent upon the basic type of CHO cell used, specific characteristics of the generated sub-clones, and type of product (Rodrigues et al. 2012; Reinhart et al. 2015). Thus, there is no single basal medium or feed suitable for all CHO cultures.

A problem with media selection is that different CHO cell types and clones have different nutrient requirements and therefore for each clone candidate the medium and feed formulation and feeding strategies would have to be screened, designed and optimized. Generally this information is not disclosed by manufacturers, which makes the early process development difficult for start-ups. Some studies were conducted and reported to select or compare commercial media for CHO cell cultures. Rodrigues et al. (2012) have compared viable cell concentration and antibody production in seven commercially available media in batch cultures of a CHO-K1 cell line and stressed the importance of selecting the correct medium for the outcome of a process. Reinhart et al. (2015) compared cell growth, antibody production, antibody quality on eight chemically defined media for a CHO DG44 cell line in batch, and with a subset of conditions in fed-batch.

To investigate how differences between clones derived from the same parental cell line may affect the choice of basal media and feeds in fed batch processes. This study investigates the effects of different basal media and feeds on cell growth, metabolism, physiology, and mAb production for two differentially behaving clones derived from the same parental cell line. The two clones were compared in 12 basal medium-feed combinations, resulting in total 24 fed-batch culture conditions. Each clone was best supported by a different basal medium-feed combination, even though they are derived from the same parental cell line. Clear metabolic shifts are observed for all the cultures when going from the growth phase to the stationary phase. Also the type and extent of these metabolic shifts are different for the 2 clones for the different basal medium-feed combinations used. Cultures fed by Actifeed A/B showed a marked increase in cell volume, which also was different for both clones being respectively threefold for BC-P and twofold for BC-G. Further improvements of cell growth and volumetric productivity by tailoring feed composition and/or feeding strategy based on specific cellular requirement are discussed.

## Material and methods

### Cell line and culture medium

Two Chinese Hamster Ovary (CHO) BC^®^ (Provided by Bioceros Holding BV, Utrecht, The Netherlands) suspension cell clones with different growth and production patterns being BC-G (high maximum cell density, low productivity) and BC-P (low maximum cell density, high productivity) were used. Both clones were derived from the same parental cell line by the same transfection and cell line generation program, and produce the same recombinant human monoclonal antibody (mAb), immunoglobulin G1 (IgG1). Each clone contained two constructs: one with a heavy chain and one with a light chain, where each construct had a different resistance marker allowing for a double selection.

Four chemically defined basal media were compared being: 1. CD FortiCHO™ (Forti, or FortiCHO) Medium, 2. CD OptiCHO™ (Opti, or OptiCHO) Medium, 3. CD-CHO Medium, and 4. ActiCHO-P (Acti, or ActiCHO) medium, with media 1, 2 and 3 purchased from Life Technologies (Bleiswijk, The Netherlands) and medium 4 purchased from GE Healthcare (Eindhoven, The Netherlands). In combination with these basal media, three chemically defined feed systems were compared being: 1. Efficient Feed™ A and B (EFA/B) from Life Technologies added in a ratio of 1:1 (v/v), based on supplier’s information; 2. Efficient Feed™ C (EFC) from Life Technologies; and 3. ActiCHO Feed™-A and -B (Acti A/B, or Actifeed A/B) from GE Healthcare added in a ratio 10:1 (v/v), based on supplier’s information. All basal media were supplemented with 4 mM l-glutamine (Gibco^®^, Life Technologies) and 0.5% (v/v) Anti-clumping agent (Gibco^®^, Life Technologies). All possible combinations of these media and feeds (3 × 4) were compared for the two CHO^BC®^ clones in fed-batch cultures.

### Pre-culture and fed-batch culture

One ampoule of cells from a working cell bank was thawed in FortiCHO medium in a 125 mL un-baffled shake flask (VWR, Radnor, PA, USA) with a 12 mL working volume. Next, the thawed culture was washed with fresh FortiCHO medium and sub-cultured in the four different basal media supplemented with selection reagents (200 µg/mL Zeocin™ and 5 µg/mL blasticidin, both from Life Technologies). Cells were sub-cultured every 3 days (1 passage) in the exponential growth phase to an initial density of 2 × 10^5^ cells/mL for at least five passages and considered adapted when they had a stable specific growth rate (µ) for two consecutive passages. Just before inoculation of the fed-batch cultures, cells were spun down at 300 × g. Spent medium was discarded and cells were re-suspended in fresh medium without selection reagents. The actual fed-batch culture experiments were done in 250 mL shake flasks (VWR) with a 25 mL initial working volume in duplicate at a seeding density of 2 × 10^5^ cells/mL. Cultures were incubated with 100 rpm shaking speed and 50 mm orbital shaking diameter in an incubator (Multitron CO_2_ incubator; Infors HT, Bottmingen, Switzerland) operated with 8% CO_2_ at 37 °C and 90% humidity. Feed addition started from culture day 3 on for all the tested conditions with a daily bolus addition. The determination of feed supply rate is explained in “Feed supply” section. Cultures were harvested on day 14, or when viability dropped below 60%.

### Cell density and viability determination

Samples were taken daily starting from culture day 2. A 1 mL sample was taken from each culture flask for measurements of total cell density, viable cell density and cell diameter using an Automated Cell Counter (TC20™; BIO-RAD, Veenendaal, The Netherlands) by trypan blue dye (Sigma-Aldrich, Zwijndrecht, The Netherlands) exclusion method. Next, the sample was filtered through a 0.2 µm filter (Minisart, Sartorius™, Rotterdam, The Netherlands). The cell free flow-through was stored at −20 °C for later analysis.

### Culture spent medium, osmolality and product titer analysis

Glucose and lactate concentrations were daily measured using an YSI analyzer (YSI 2700, YSI Life Sciences, Yellow Springs, OH, USA). Ammonium concentrations were analyzed by a BioProfile^®^ FLEX analyzer (Nova biomedical, Waltham, MA, USA) after the experiment was finished. Spent medium composition including all the extracellular amino acids was quantified using NMR (Spinnovation biologics BV, Oss, The Netherlands) based on the sample scheme (Table 1) after the experiment was finished. IgG concentration was determined by Bioceros Netherlands BV, using the Octet system with protein G biosensors (ForteBio, Pall, Portsmouth, UK). Osmolality was measured using an Osmomat 030-D cryoscopic osmometer (Gonotec, Berlin, Germany).

**Table 1:** Sample points for spent media analysis. Samples for spent media analysis were carried out on every other day for each culture. Grey cells: culture was terminated

Day	0	2	4	6	8	10	12	13	14
BC-P									
Opti + EFA/B	√	√	√	√	√	√			
Opti + Acti A/B	√	√	√	√	√	√	√		√
Acti + EFA/B	√	√	√	√	√	√	√	√	
Acti + Acti A/B	√	√	√	√	√	√	√		√
BC-G									
Opti + EFA/B	√	√	√	√	√	√	√		√
Opti + Acti A/B	√	√	√	√	√	√	√		
Acti + EFA/B	√	√	√	√	√	√	√		√
Acti + Acti A/B	√	√	√	√	√	√	√		√

### Productivity

Based on cell growth patterns of fed-batch cultures, four culture phases were distinguished:

1. Start-up phase (L-Phase), from the inoculation day until day 2;
2. Exponential growth phase (G-phase), from day 2 until the day that the cultures reached the maximum cell density;
3. Stationary phase (S-Phase), from the end of G-Phase until the culture viability was lower than 80%; and
4. Death phase (D-Phase), is from the end of S-Phase until the end of the culture.

This study mainly focuses on the G- and the S-phase during the cultures. The calculation of average specific consumption/production rates was done separately for the growth and stationary phase (Fig. 8).

Change in the total amount of compound x in the shaker flask is given by

1 $$\frac{{dM_{x} }}{dt} = q_{x} \cdot M_{VC} \left( t \right)$$

where M_x_ (mg; mmol) is the total amount of compound x in a culture, M_VC_ (cells) is the number of viable cells in a culture and q_x_ (mmol·cell^−1^·day^−1^) is the specific production rate of compound x. With constant q_x_, integration of Eq. 1 gives:

2 $$M_{x} \left( t \right) - M_{x} \left( 0 \right) = q_{x} \cdot \mathop \smallint \limits_{0}^{t} M_{VC}$$

The average specific production rates of glucose, essential amino acids and antibody were obtained from a plot of the total amount of a compound against the integral of viable cell numbers using linear regression (Appendix 1 of ESM). Positive values indicate production while negative values indicate consumption.

### Feed supply

Glucose serves as one of the main carbon and energy sources for CHO cells (Zamorano et al. 2010). The consumption rate of glucose is often used as an indicator of cell culture activity to guide feeding strategies. Since the specific consumption rate of glucose at per cell level does not vary much if cell activity is unchanged and glucose concentrations are well above the Monod saturation constant. In this study, it was not possible to conduct daily measurement for nutrients other than glucose and lactate. The daily amount of feed addition, therefore, was based on the glucose concentration at the moment of feeding and the anticipated glucose consumption rate for the next 24 h according to glucose specific consumption rate of the cells. The feeding strategy used in this study is different from the supplier’s protocol. However, since the protocols differ among suppliers, the feeding strategy of this study gives fairer comparison between different feed systems. The feeding strategy presumes that the glucose concentration in the feed is well balanced with the rest of the nutrients. To calculate the daily feed amount, the following method was used. In addition, an example calculation is shown in Appendix 2 of ESM.

First, the specific growth rate µ_*g*_ (day^−1^) is calculated by

3 $$\mu_{g} = \frac{{\ln \frac{{VCD_{Y} }}{{VCD_{Y - 1} }}}}{{t_{Y} - t_{Y - 1} }}$$

where VCD_Y−1_ and VCD_Y_ (cells·mL^−1^) are the viable cell densities of the last and the current feeding points, respectively. t_Y−1_ and t_Y_ (day) are the time points for the last and the current feeding points, respectively. Then, the VCD of the next day can be predicted based on Eq. 4.

4 $$VCD_{Y + 1} = VCD_{Y} \times e^{{\mu_{g} \cdot \left( {t_{Y + 1} - t_{Y} } \right)}}$$

Next the integral viable cell density (IVCD) of the previous and the next culture day was calculated by Eq. 5 and Eq. 6 respectively.

5 $$\Delta IVCD_{Y} = VCD_{Y - 1} \times \left( {t_{Y} - t_{Y - 1} } \right) + \frac{{\left( {VCD_{Y} - VCD_{Y - 1} } \right) \times \left( {t_{Y} - t_{Y - 1} } \right)}}{2}$$

6 $$\Delta IVCD_{Y + 1} = VCD_{Y} \times \left( {t_{Y + 1} - t_{Y} } \right) + \frac{{\left( {VCD_{Y + 1} - VCD_{Y} } \right) \times \left( {t_{Y + 1} - t_{Y} } \right)}}{2}$$

IVCD was defined as the area under the viable cell growth curve using the trapezium rule. ΔIVCD_Y_ and ΔIVCD_Y+1_ (cells·day·mL^−1^) are the IVCD of the past day and the predicted next day, respectively.

The specific glucose consumption rate ($q_{{{\text{Gluc}}_{\text{Y}} }}$, mmol·cell^−1^·day^−1^) can then be calculated by:

7 $$\frac{{\Delta GLC_{Y} }}{{\Delta IVCD_{Y} }} = q_{{Glc_{Y} }}$$

Assuming the specific glucose consumption rate stays constant, the glucose consumption during the next day (ΔGLC_Y+1_, mM) can then be predicted by:

8 $$\Delta GLC_{Y + 1} = q_{{Glc_{Y} }} \times \Delta IVCD_{Y + 1}$$

Finally, the feed volume to be added was determined by using Eq. 9. Glucose concentration was kept at 10 mM (as a safe threshold) by the next day sample point.

9 $$V_{F} = \frac{{(\Delta GLC_{Y + 1} + \left( {10 - GLC_{Y} } \right)) \times V_{Y} }}{{C_{F} }}$$

where GLC_Y_ is the current glucose concentration (mM), V_F_ is the feed volume to be added (mL), V_Y_ is the current culture volume (mL) and C_F_ is the glucose concentration in the feed (mM).

For each duplicate culture the feeding volume was calculated. Next the two obtained values were averaged and the average feeding volume was added to both cultures. By using this feeding method, we managed to keep the range of glucose between 10 and 30 mM throughout the fed-batch cultures by daily bolus feeding.

### Balance of essential amino acids in feeds

To check whether the essential amino acid and glucose concentrations in the feed are balanced with respect to the needs of the cells, a relative balancing level is defined

10 $${\text{Relative balancing level }} = \left( {\frac{{\frac{{C_{{EAA_{x} }} }}{{C_{glc} }}}}{{\frac{{q_{{EAA_{x} }} }}{{q_{glc} }}}}} \right)$$

where *C*_*EAAx*_ and *C*_*glc*_ (all in mM) are concentrations of essential amino acid x and glucose in the feed, *q*_*EAAx*_ and *q*_*glc*_ (all in mmol·cell^−1^·day^−1^) are the specific consumption rates of essential amino acid x and glucose during the growth phase or the stationary phase of the culture. In this way, the ratio of essential amino acid x to glucose is compared between the formulation of feed and the cell specific consumption rates. Since the feed supply rate was based on glucose concentration in this experiment, the feed balance of essential amino acids was evaluated based on glucose. A relative balancing level equal to 1 means that the rate with which the essential amino acid is supplied by the feed equals its consumption rate when feed addition is based on glucose. A value other than 1 means that the supply rate of the essential amino acid is either lower (<1) than or higher (>1) than the specific consumption rate, resulting in either depletion or accumulation.

## Results

### Media and feed compositions

**Fig. 1:**
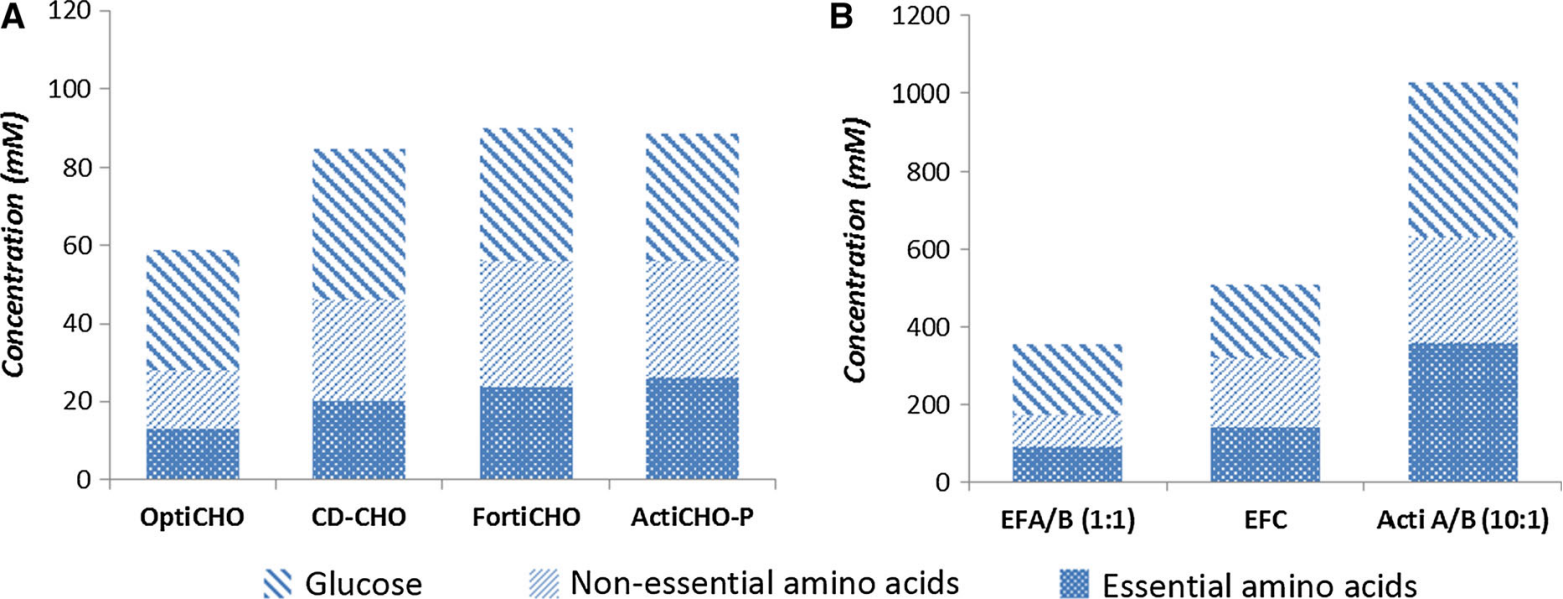
Sum of concentrations of non-essential amino acids, of essential amino acids, and concentration of glucose in different basal media (**a**) and feeds (**b**). EFA and EFB are used in a ratio of 1:1 (v/v) and Actifeed A/B in a ratio of 10:1 (v/v)

The composition of the used basal media and feeds was measured with respect to amino acids and glucose (Fig. 1). An excel file of the measured compositions is listed in Appendix 4 of ESM. All basal media and feeds contain glucose, non-essential amino acids and essential amino acids. In general, the feeds are 5–10 times more concentrated than the basal media in both glucose and amino acids. For the basal media, OptiCHO is relatively poorer whereas FortiCHO and ActiCHO-P are richer in amino acids content. For the feeds, Actifeed A/B is richer than EFA/B (threefold) and EFC (twofold) in amino acids and glucose content.

### Performance of BC-P and BC-G clones in different medium-feed combinations

**Fig. 2:**
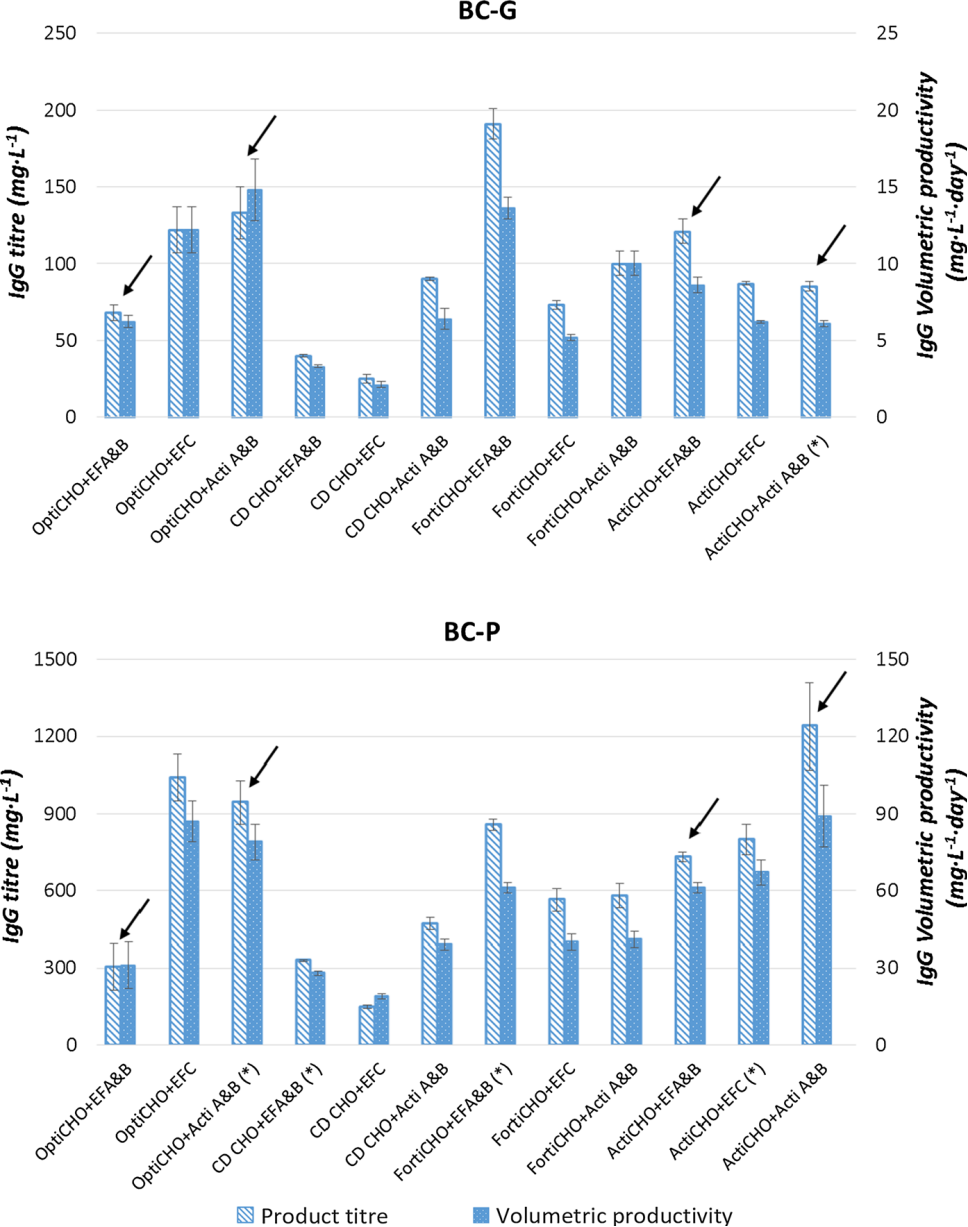
Product titer ( mg L^**−**1^) and volumetric productivity ( mg L^**−**1^ day^−1^) of clone BC-G and BC-P for all tested basal medium-feed combinations. *Error bars* indicate the deviation of the duplicate data points from the average value. Conditions marked with *arrows* were selected for further comparisons. Conditions marked with *asterisks* indicate cell aggregation during the cultures

Volumetric productivity and product titer of the mAb are the most often used parameters during media and clone selection in the early culture process development phase. Therefore the volumetric productivity and IgG product titer of all the studied conditions were compared (Fig. 2). For the BC-G clone, FortiCHO + EFA/B resulted in the highest titer (190 mg L^−1^) and volumetric productivity (14  mg L^−1^ day^−1^). For the BC-P clone, ActiCHO + Actifeed A/B resulted in the highest titer (1250  mg L^−1^) and volumetric productivity (90  mg L^−1^ day^−1^). Overall, the BC-P clone showed clearly broader ranges (up to sixfold higher) of IgG titer and volumetric productivity compared to the BC-G clone. Compared to the other basal media, the use of CD-CHO resulted in the lowest volumetric productivities and product titers for both clones irrespective of the feed used. Other output parameters including maximum viable cell density, integral viable cell density, specific growth rate, number of days where the viability is above 80% and specific productivity can be found in Appendix 3 of ESM.

**Fig. 3:**
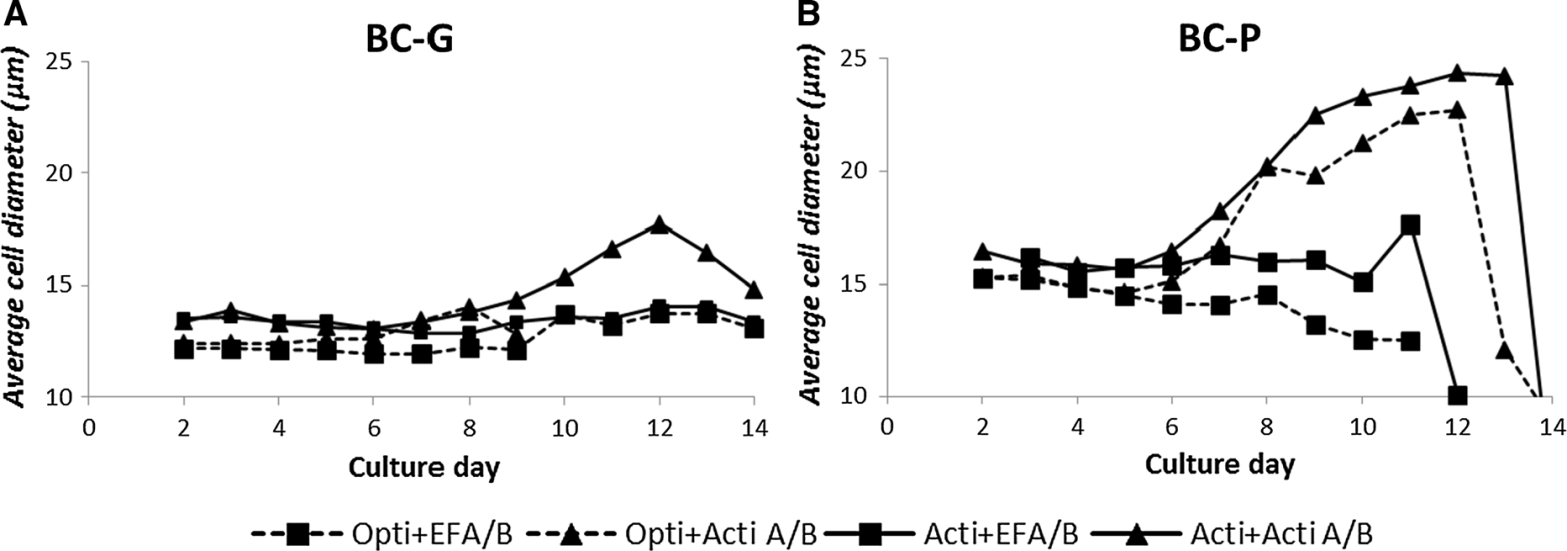
Average cell diameters (µm) of the BC-G (**a**) and BC-P (**b**) clones in the selected fed-batch culture conditions

Cell physiology responded differently to medium-feed combinations. Remarkably, for both clones when fed by Actifeed A/B, an increase of cell diameter during the stationary phase was observed. The increase was not observed when the other feeds (EFA/B and EFC) were used (data in Fig. 3 only shows EFA/B). For the BC-P clone the size increase was most prominent with an increase of the average cell diameter from 16 to 24 µm (Fig. 3), which is equivalent to a cell volume increase of nearly threefold. The cell size increase was also observed for the BC-G clone fed by Actifeed A/B, but to a lesser extent being from 14 to 18 µm. For the culture using Opti + Acti A/B with the BC-G clone in Fig. 3, the cell size increase could not be observed because it was terminated on day 9 due to a low viability. The observation implies a common response between the two clones towards the feed addition. The sudden decrease of cell diameter at the end of the cultures was due to cell death.

### Metabolite profiles in fed-batch cultures

To study the effect of different medium-feed combinations on cell metabolism, 4 combinations (marked with arrows in Fig. 2) were selected for further comparison being: a relatively poor medium (OptiCHO) with a poor feed (EFA/B) and a rich feed (Actifeed A/B) and a rich basal medium (ActiCHO) with the same feeds. In this way, the impact of the richness of media and feeds was compared in more detail. Amongst these conditions the cultures in which the highest volumetric productivities were obtained (BC-G in OptiCHO + Actifeed A/B and BC-P in ActiCHO + Actifeed A/B) are included. For these conditions spent media analysis was conducted at different time points according to Table 1. Since cells grown on FortiCHO + EFA/B and OptiCHO + EFC also gave high titers for both clones, their spent media were also analyzed. The results are listed in Appendix 4 of ESM.

**Fig. 4:**
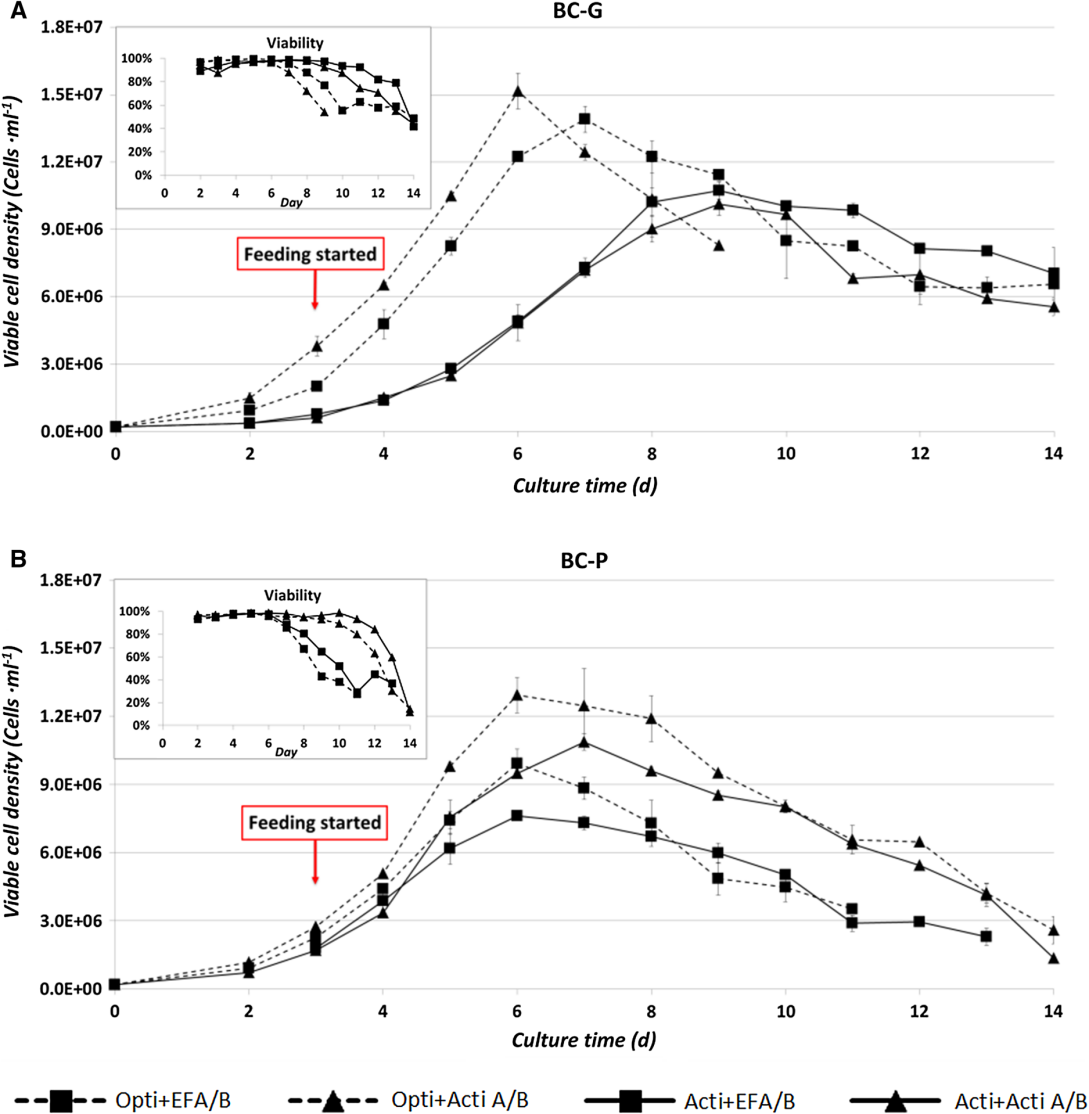
Viable cell density and viability of the BC-G (**a**) and BC-P (**b**) clones in the selected conditions. *Error bars* show the deviation of the duplicate data points from the average value

Figure 4 shows the viable cell density and viability of the selected culture conditions. For the BC-G clone, the growth patterns were similar when using the same basal medium, while for the BC-P clone, the growth patterns were similar when using the same feed. In all medium-feed combinations, cell division stopped after culture day 6, except for the BC-G clone in ActiCHO medium, where cell division lasted until day 8 but at a lower maximum specific rate of ~0.55/day compared to ~0.70/day for the BC-G clone in OptiCHO medium (Appendix 3 of ESM). The decline of viable cell density during the stationary phase was caused by dilution due to sampling and feeding, and/or due to cell death.

#### Glucose, lactate, ammonium and osmolality profiles

**Fig. 5:**
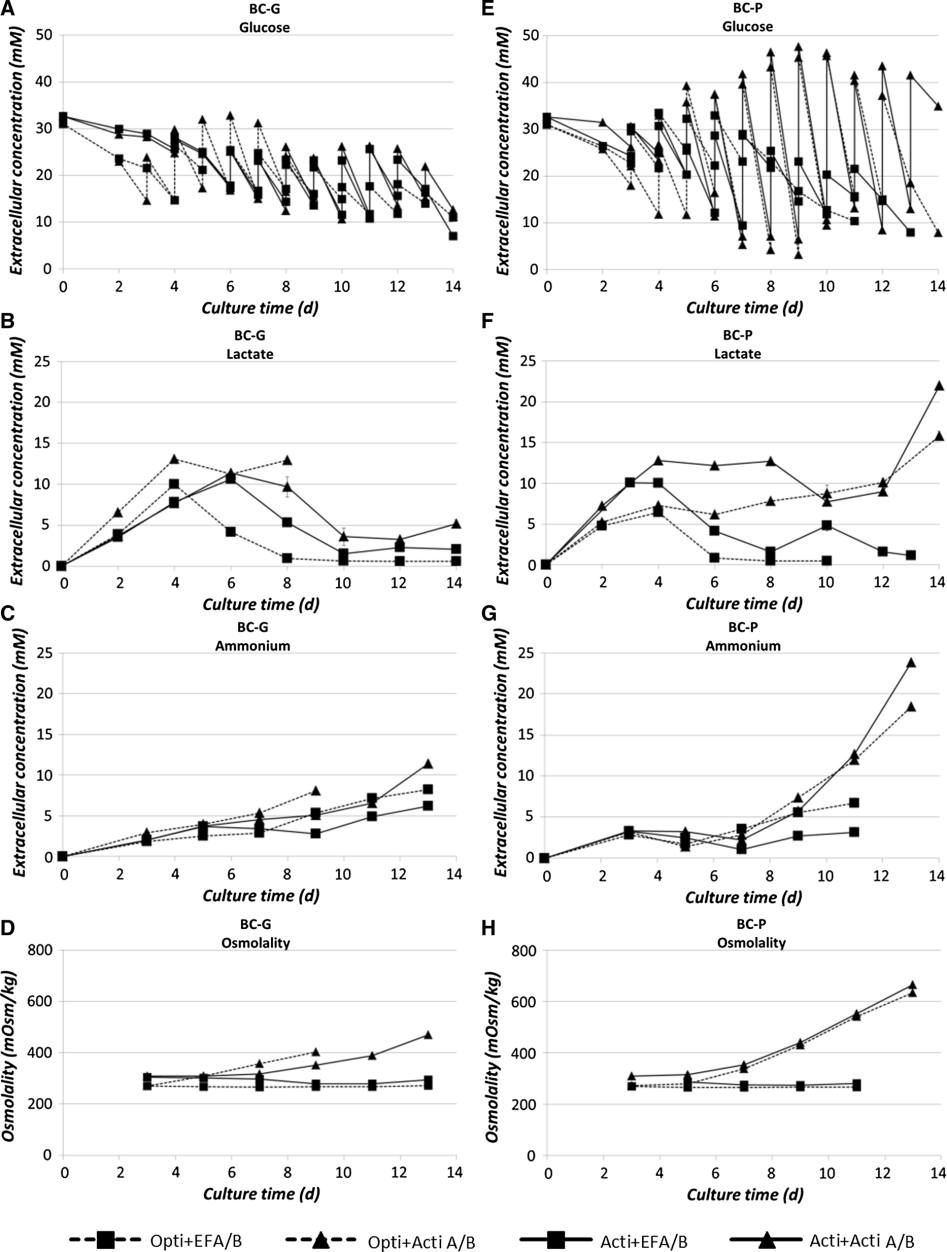
Extracellular concentration of glucose, lactate, ammonium (Graph **a**, **b**, **c**, **e**, **f** and **g**: unit in mM) and culture osmolality (Graph **d** and **h**: unit in mOsm/kg) for the two clones during fed-batch cultures. *Error bars* show the deviation of the duplicate data points from the average value

Glucose shows similar zigzag profiles for the tested conditions due to consumption and daily feed addition starting from day 3 on (Fig. 5a, e). For the BC-P clone fed by Actifeed A/B during the stationary phase, a considerably higher daily glucose consumption is observed (Fig. 5e). For both clones the lactate concentration shows a similar trend with an initial increase followed by a decrease after 3–4 days except again for the BC-P clone fed by Actifeed A/B, where the lactate concentration kept increasing until the end of the culture. The observed simultaneous consumption of lactate and glucose agrees with literature on various other CHO cell lines (Zagari et al. 2013; Li et al. 2012; Mulukutla et al. 2012). Ammonium levels keep increasing during all fed-batch cultures. For the BC-P clone fed with Actifeed A/B, clearly higher ammonium concentrations are observed (Fig. 5c, g). The osmolality is constant except for the cultures fed by Actifeed A/B, where an increase up to 400 and 620 mOsm/kg is observed for respectively the BC-G and the BC-P clone (Fig. 5d, h). Both clones seem to cope well with the osmolality since the viability maintained higher than 80% until day 12.

#### Essential amino acid profiles

**Fig. 6:**
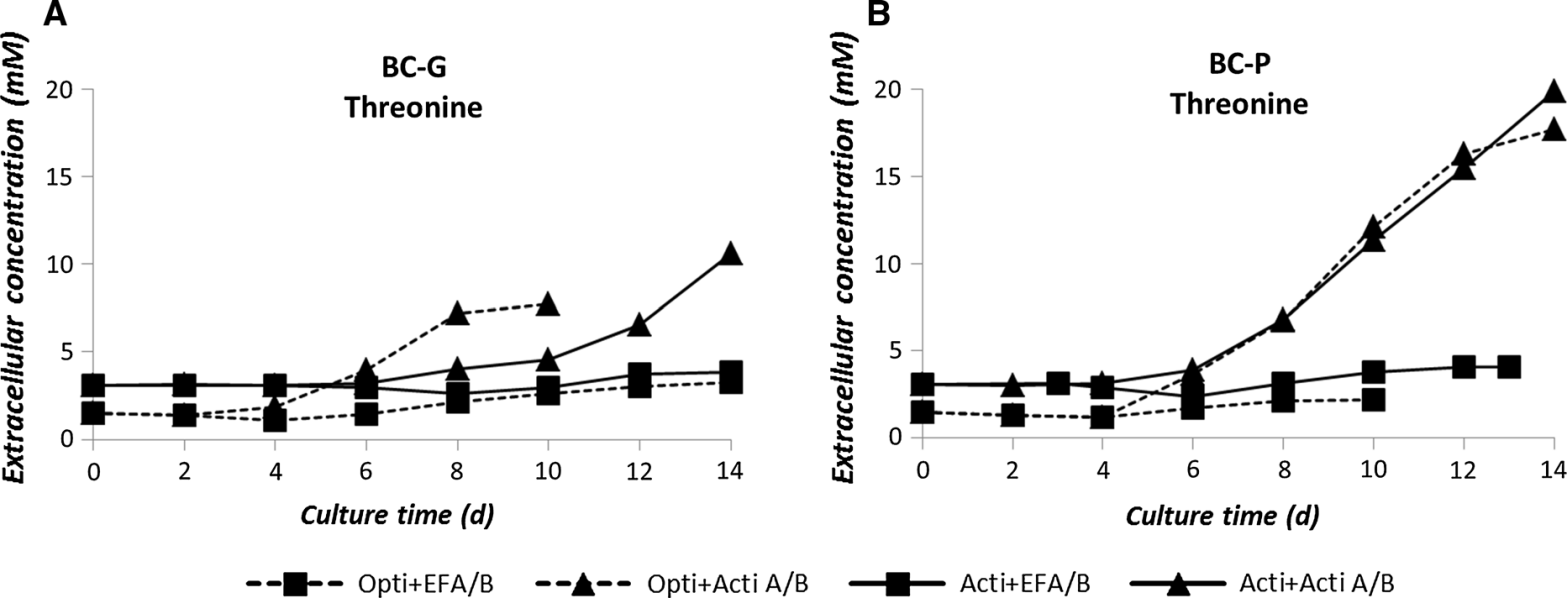
Extracellular threonine concentrations (mM) during fed-batch cultures of the both clones. Error bars show the deviation of the duplicate data points from the average value

In each culture condition, the essential amino acid profiles show similar trends. As an example, threonine concentrations of the selected culture conditions are shown (Fig. 6). The other individual essential amino acids including valine, isoleucine, leucine, lysine, methionine, tryptophan, histidine and phenylalanine are listed in Appendix 4 of ESM. None of the essential amino acids was depleted during fed-batch cultures, indicating that they were not the limiting nutrients for cell growth or production in all studied cases. Cultures fed by EFA/B resulted in stable essential amino acid concentrations, whereas cultures fed by Actifeed A/B resulted in accumulation of essential amino acids starting from the stationary phase (day 6). The accumulation was larger for the BC-P clone than for the BC-G clone.

#### Non*-*essential amino acid profiles

**Fig. 7:**
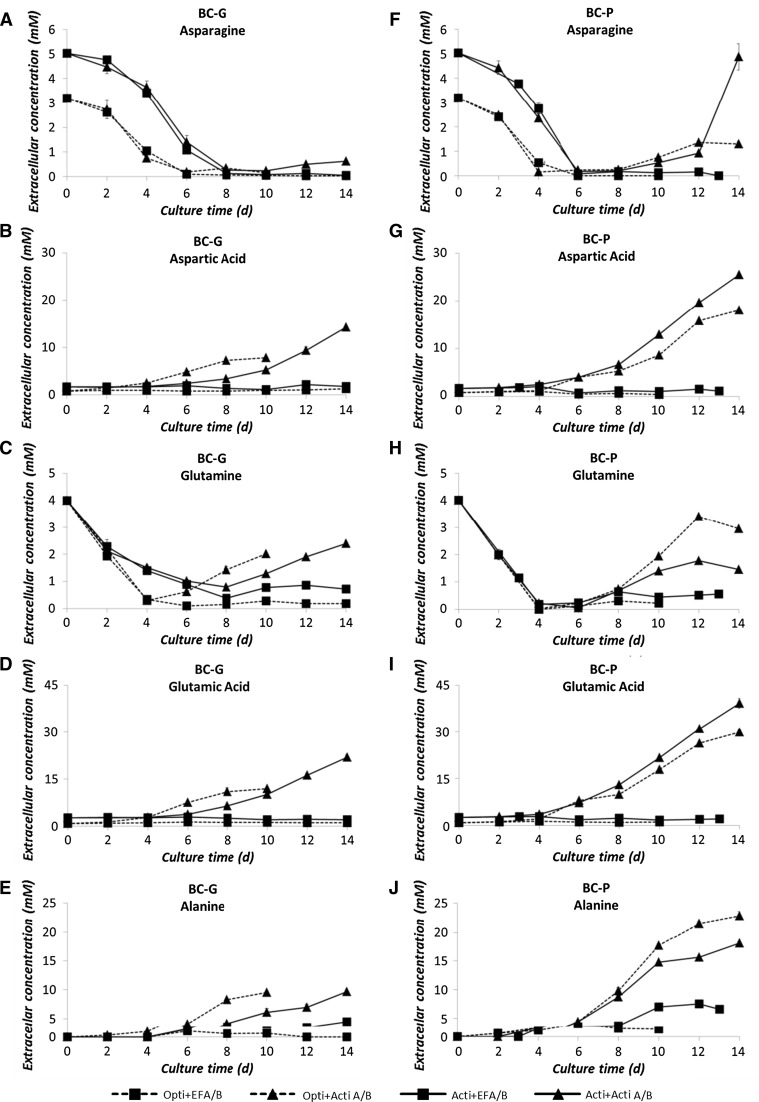
Non-essential amino acid profiles of selected culture conditions in fed-batch cultures. Concentrations are shown in mM. Graph **a**, **b**, **c**, **d**, **e** show the profiles of asparagine, aspartic acid, glutamine, glutamic acid and alanine of the BC-G clone; Graph **f**, **g**, **h**, **i**, **j** show their profiles of the BC-P clone. Error bars show the deviation of the duplicate data points from the average value

Figure 7 shows the profiles of asparagine, aspartic acid, glutamine, glutamic acid and alanine, which are the non-essential amino acids directly related to the primary carbon metabolism. The other measured amino acids are shown in Appendix 4 of ESM. In most of the conditions, asparagine and glutamine were depleted between day 4 and day 6 (Fig. 7a, c, f, h). When using Actifeed A/B feed, glutamine, which is not present in the feed, was produced in the later culture stage as indicated by the increased glutamine concentration. The sudden increase of asparagine concentration at day 14 in Fig. 7f was most likely a measurement error, since it was not observed throughout other cultures. Aspartic acid and glutamic acid stayed at low concentrations during EFA/B fed cultures, but increased during Actifeed A/B fed cultures (Fig. 7b, d, g, i), because of the high content of these two compounds (~50 and ~70 mM respectively) in Actifeed A/B. Alanine is not present in the basal media, but is present in EFA/B feed (~1 mM) and Actifeed A/B (~16 mM). As seen in Fig. 7e, j, all selected culture conditions showed an increase of alanine concentration except for the poor medium and poor feed (OptiCHO + EFA/B) where alanine was consumed after day 6. In the cultures with the rich medium and poor feed (ActiCHO + EFA/B), the alanine increase was mostly due to production by cells, whereas when fed by the rich feed (Actifeed A/B) the increase was due to both feeding and production. When fed by feed EFA/B, depletions of tyrosine and cystine (Appendix 4 of ESM) were observed at around day 6 for both the clones.

### Specific consumption rates

**Fig. 8:**
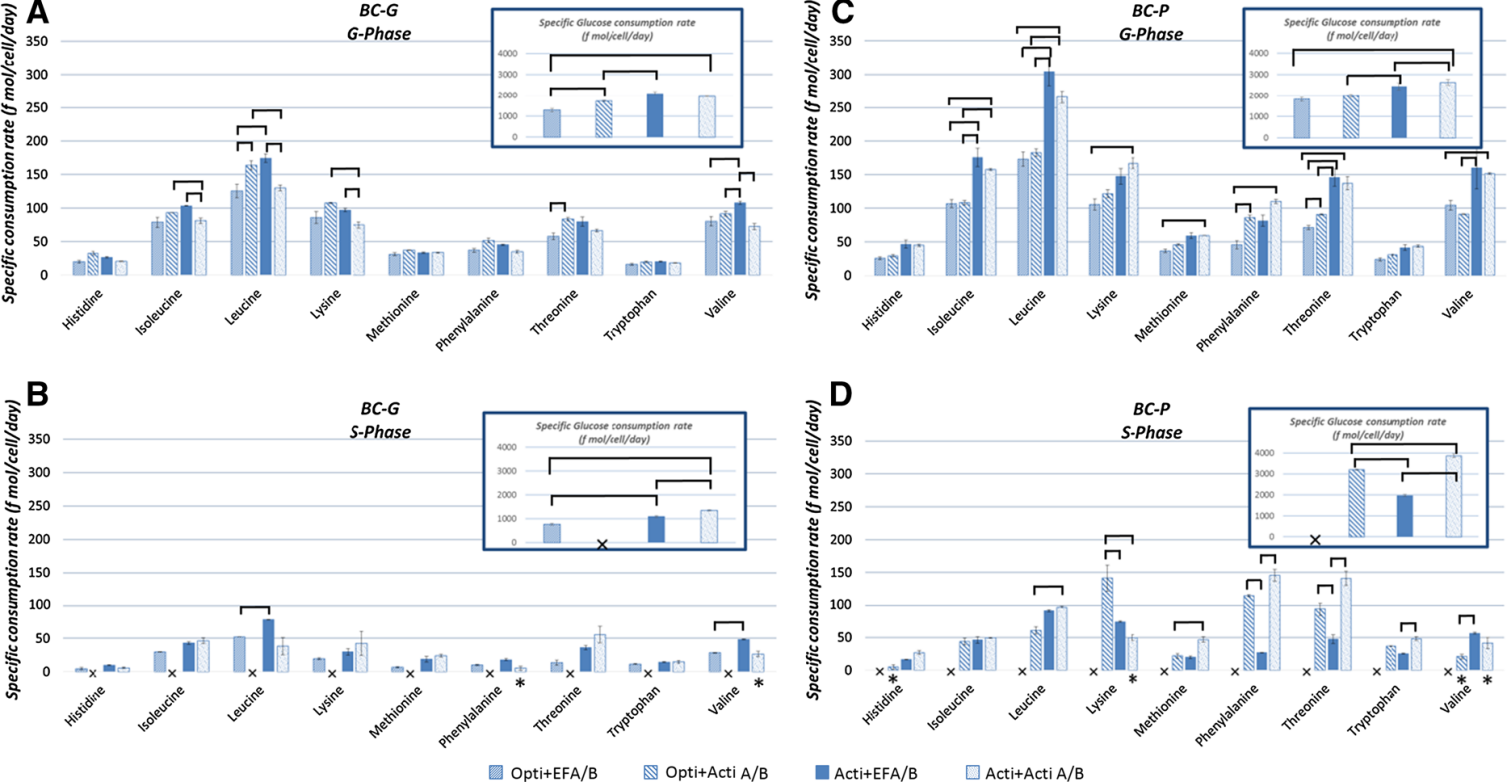
Average specific consumption rates (f fmol·cell^**−**1^ day^−1^) of the 9 essential amino acids and glucose of the BC-G (**a**, **b**) and the BC-P (**c**, **d**) clones in the selected fed-batch culture conditions. *G-Phase* growth phase; *S-Phase* stationary phase. Average specific consumption rates of the conditions indicated with (×) and (*) cannot be calculated due to early cell death and inaccurate (R^2^ < 0.8) specific consumption rate calculations, respectively. “” shows a significant (*P* < 0.05) difference between the linked two values

**Fig. 9:**
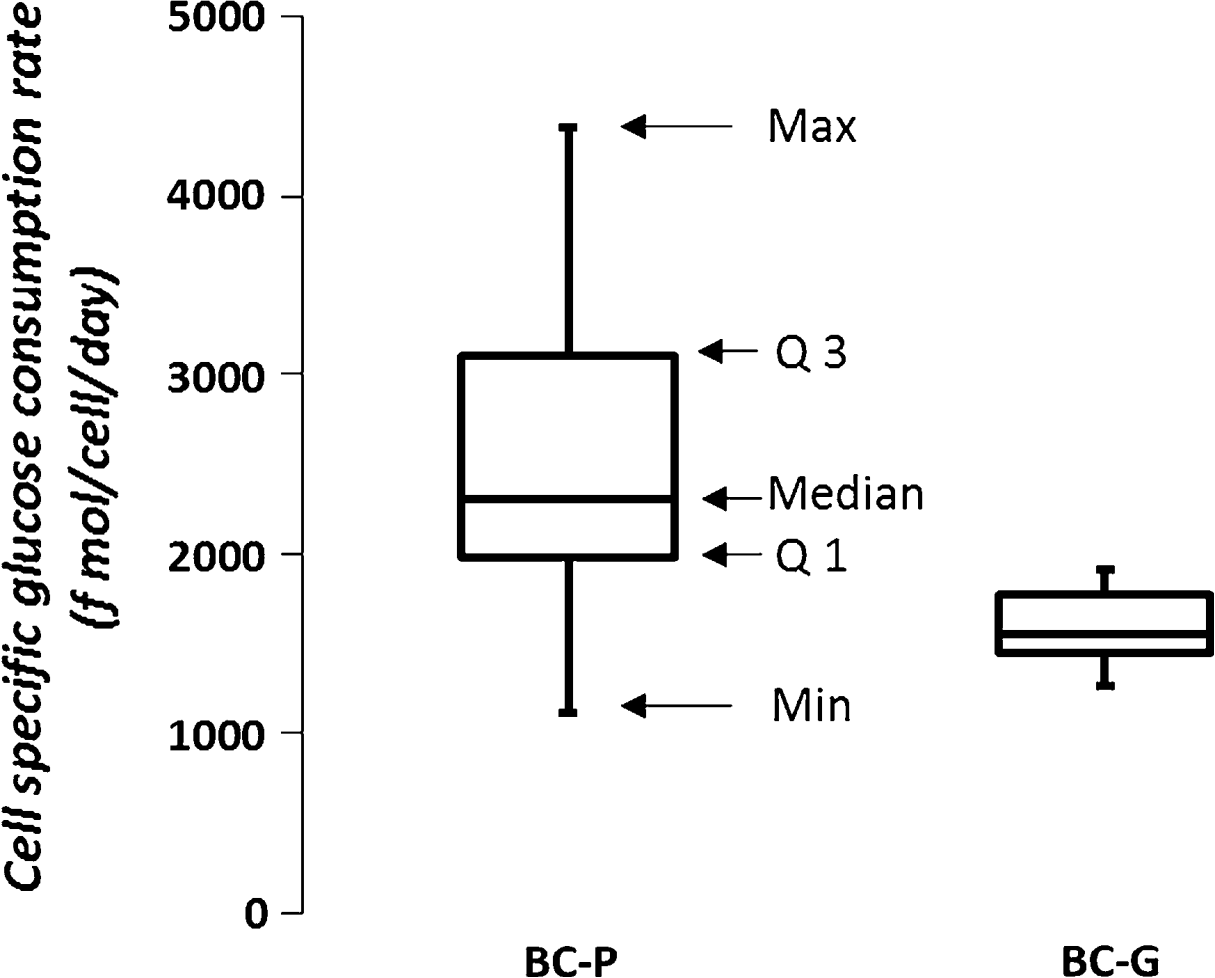
Overall cell specific glucose consumption rates (f mol/cell/day) for the BC-P and BC-G clone ranges observed in all the tested conditions. The maximum (Max), minimum (Min), median, quartile 1 (Q1), and quartile 2 (Q2) values are shown by arrows for the BC-P clones; same follows for the BC-G clone

In a fed-batch culture, the essential amino acid feeding rate should match the consumption rates of the cells. The average specific consumption rates of essential amino acids and glucose are presented during the growth and the stationary phase for both clones (Fig. 8). Note that on average, the ratios among essential amino acids for two clones are similar during the growth phase, indicating similar requirements for both clones to make biomass during this phase. In general, average specific consumption rates of most of the essential amino acids during the growth phase are significantly (*T* tests, data not shown) higher than during the stationary phase. This is also true for the average specific consumption rate of glucose, except for the BC-P clone fed by Actifeed A/B, where the average specific glucose consumption rate is higher in the stationary phase than in growth phase (Fig. 8c, d). Overall, the BC-P clone shows higher average specific glucose and essential amino acids consumption rates compared to the BC-G clone, which may be related to the larger cell size of the BC-P clone (~16 µm) compared to the BC-G clone (~13 µm) (Fig. 3). In order to show the variation of specific glucose consumption rate during, a boxplot of the specific glucose consumption rate for all the tested conditions during different culture phases is shown in Fig. 9. The specific glucose consumption rate varies in a wide range in different media/feed conditions for the BC-P clone, whereas in a much smaller range for the BC-G clone.

## Discussion

### Effects of media and feed on culture performances and cell physiology

In total, 12 combinations of four basal media and three feeds were tested for two CHO daughter clones. As expected, the BC-P clone reached higher volumetric productivities and product titers. Overall, no clear correlation between richness level of the media or feed and the performance of the clones was observed. For example, the BC-G clone reached the highest titer (191  mg L^−1^) in FortiCHO + EFA/B, which is a rich medium with a poor feed, while for this clone ActiCHO with Actifeed A/B, which is a rich medium with a rich feed performed clearly poorer (Fig. 2). For both clones, CD-CHO medium did not support mAb production. Notably, even though the 2 clones are generated from the same parent and produce the same mAb, they do not share a common medium-feed for their optimal mAb production.

It was observed that the addition of Actifeed A/B resulted in a cell volume increase for both the clones with a threefold increase for the BC-P clone and a twofold increase for the BC-G clone. The increase of cell size did not occur when fed by EFA/B and EFC, which indicates that the cell size increase is not an intrinsic property of the cell line, but rather a physiological response to the feed system. Several possibilities might cause the size increase: 1. Cell cycle arrest. It was reported that overexpression of a cell cycle inhibitor (p21^CIP1^) in a CHO cell line resulted in a fourfold increase of cell volume (Bi et al. 2004) For overexpression of another inhibitor (p27) in a CHO cell line a twofold increase of cell protein content was measured (Carvalhal et al. 2003). When cells are arrested at the G1 phase, they continue to grow in size instead of passing the dividing checkpoint. It might be that in this study the depletion of certain nutrients has led to a cell cycle arrest. Note that there are still many nutrients (e.g. vitamins and trace elements) in the basal media and feeds that were not measured in this study. 2. Another possibility could be that the overfed nutrients induced cell cycle arrest. The overfeeding resulted in higher rate of cellular transportation of amino acids, the accumulation of these precursors triggers the higher formation rate of biopolymers. Oh et al. (1995) demonstrated an increase of amino acid uptake via Na^+^-dependent transport systems caused by elevation of osmolality. 3. It might also be that the cells get larger by taking up water. However, this is typically seen when cells are exposed to hypotonic pressure (Hoffmann et al. 2009). Furthermore, it was observed that a higher average mAb specific productivity is correlated with conditions that result in a larger cell size (the BC-P clone fed by Actifeed A/B; Appendix 3 of ESM). Cell specific mAb productivity has been reported to correlate with cell volume (Bi et al. 2004; Lloyd et al. 2000; Martínez et al. 2015). Kim et al. (2001) suggested a linear correlation between the increase in specific thrombopoietin productivity and the increase of cell size for the 9 CHO sub-clones they studied. Results from this study seem to agree with their observation. However, this experiment is unable to present the precise comparison of the specific mAb productivity before and after the cell size increase, since the cultures were performed in uncontrolled shake flasks with culture volumes constantly changing by sampling and feeding.

The BC-P clone tended to form aggregates in more medium-feed combinations compared to the BC-G clone (indicated by the asterisks in Fig. 2). Formation of aggregates indicates suboptimal growth conditions as aggregates were reported to be formed around decaying and dead cells through the released DNA (Renner et al. 1993). In this study, addition of 0.5% anti-clumping agent in the basal media helped to alleviate the aggregating problem, especially to the BC-P clone. The longevity of cultures, therefore, was prolonged up to 4 more days (data not shown).

### Cell metabolism and feed composition

Around day 4–6 all cultures showed a transition from the growth phase to the stationary phase, which is commonly observed for CHO cells in batch and fed-batch cultures (Mulukutla et al. 2012; Ahn and Antoniewicz 2011; Martínez et al. 2013; Young 2013). Nevertheless, the relation between overall metabolite profiles and this transition is still poorly understood, which is due to the highly complex and dynamic nature of CHO cell metabolism and the complexity of the media used. In this section, metabolite profiles are described with a focus on the changes that occurred during the culture phase transition. It is clear from “Glucose, lactate, ammonium and osmolality profiles” section that the transition from growth to stationary phase was not caused by depletion of glucose or essential amino acids. It is also not likely that the transition was caused by lactate and ammonium inhibition, since the concentrations of lactate and ammonium at the transition points were below the reported growth inhibition levels of 5–8 mM for ammonia (Hansen and Emborg 1994; Yang and Butler 2000) and 30 mM for lactate (Lao and Toth 1997). In most of the tested conditions, however, glutamine and asparagine were depleted between day 4 and day 6 (Fig. 7), which coincided with the phase transition, and a shift of lactate metabolism from net production to net consumption. For the BC-G clone cultured in ActiCHO medium, depletion of asparagine and glutamine (Fig. 7) occurred later probably as a consequence of a lower growth rate (Appendix 3 of ESM). For this culture the transition to stationary phase and the shift in lactate metabolism were also delayed (Fig. 5b). It was reported that the depletion of asparagine and glutamine coincides with CHO cell growth suppression (Fomina-Yadlin et al. 2014). On the other hand, as can be seen in this study, glutamate and glutamine were accumulated at the later stage of the cultures. Since glutamine is not present in the feed, this shows that the CHO cells used in this study can produce glutamine from glutamate and therefore glutamine is not likely limiting the cultures. Asparagine is present in the feed. Although asparagine was depleted during the later stage of the cultures, it might be converted to aspartate by cells resulting in the accumulation of aspartate (Fig. 7b, g). In the combination of OptiCHO + EFA/B, apart from asparagine and glutamine, also tyrosine and cystine (Appendix 4 of ESM) were depleted around the culture transition point for both clones, which might be related to the lower mAb productivities (Appendix 3 of ESM). Similar observations of tyrosine and cystine depletion affecting protein production were shown for CHO cells (Yu et al. 2011) and hybridoma cells (Read et al. 2013). The non-essential amino acids can be produced by cells, therefore the effects of their depletion at extracellular level to the culture performance could not be confirmed in this study. The essential amino acids however, cannot be made by cells. It is important to avoid their depletion.

Specific consumption rates of glucose and amino acids are expected to decrease when a culture shifts from the growth to the stationary phase (Ahn and Antoniewicz 2011) due to decrease of the specific growth rate. However, in contrast to all the other tested conditions, for the BC-P clone fed by Actifeed A/B, the average specific glucose consumption rate increased in the stationary phase with a factor 1.5: from 2000 to 3200 fmol·cell^−1^ day^−1^ grown in OptiCHO basal medium and from 2700 to 3900 fmol·cell^−1^ day^−1^ grown in ActiCHO basal medium (Fig. 8). The higher specific glucose consumption rate correlates with the fact that cells become bigger (only for BC-G in Opti + Actifeed A/B this is not observed, because this culture had to be stopped before the increase became apparent). It is likely that bigger cells need more energy for growth and maintenance which is supplied by higher glucose consumption rates. It is also worth noting that when the average glucose consumption rate is calculated per cell volume (data not shown), a drop is observed upon the transition from growth to stationary phase just as with the other conditions. For the BC-P clone also more lactate is formed during the stationary phase (Fig. 5f), which means even more glucose needs to be consumed, since lactate formation is less efficient for ATP generation. In addition, production of alanine for both clones was observed (Fig. 7e, j). The production was not observed when both clones were cultured in poor medium-feed combinations (OptiCHO + EFA/B). Extra carbons generated from glycolysis can also be diverted to alanine production through transaminase. This step at the same time transports amine groups out of the cells. Richer media and feeds (e.g. ActiCHO + Actifeed A/B) supply sufficient amino acid nitrogen to make all the needed alanine and thus alanine was even produced in these cases, while for the poor media and feeds (e.g. OptiCHO + EFA/B) this is not the case and additional alanine is taken up by the cells.

**Fig. 10:**
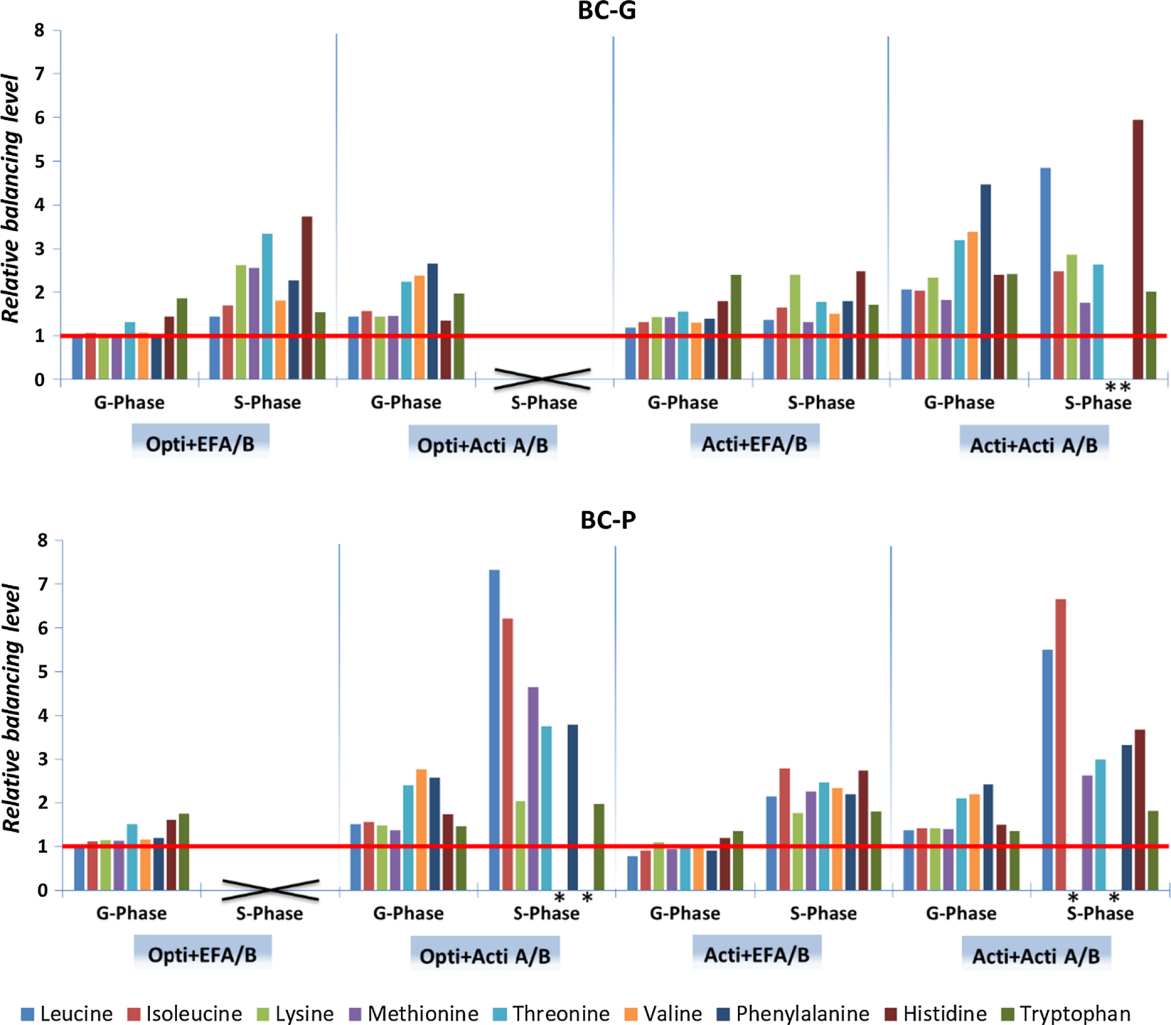
Relative feed balancing level of essential amino acids in different culture conditions during growth phase (*G-Phase*) and stationary phase (*S-Phase*). A value other than 1 means that the supply of the essential amino acid is either lower (<1) than or higher (>1) than the specific consumption rate, resulting in either depletion or accumulation. Relative balancing levels indicated with (×) and (*) cannot be calculated due to early cell death and inaccurate (R^2^ < 0.8) specific consumption rate calculations, respectively

**Fig. 11:**
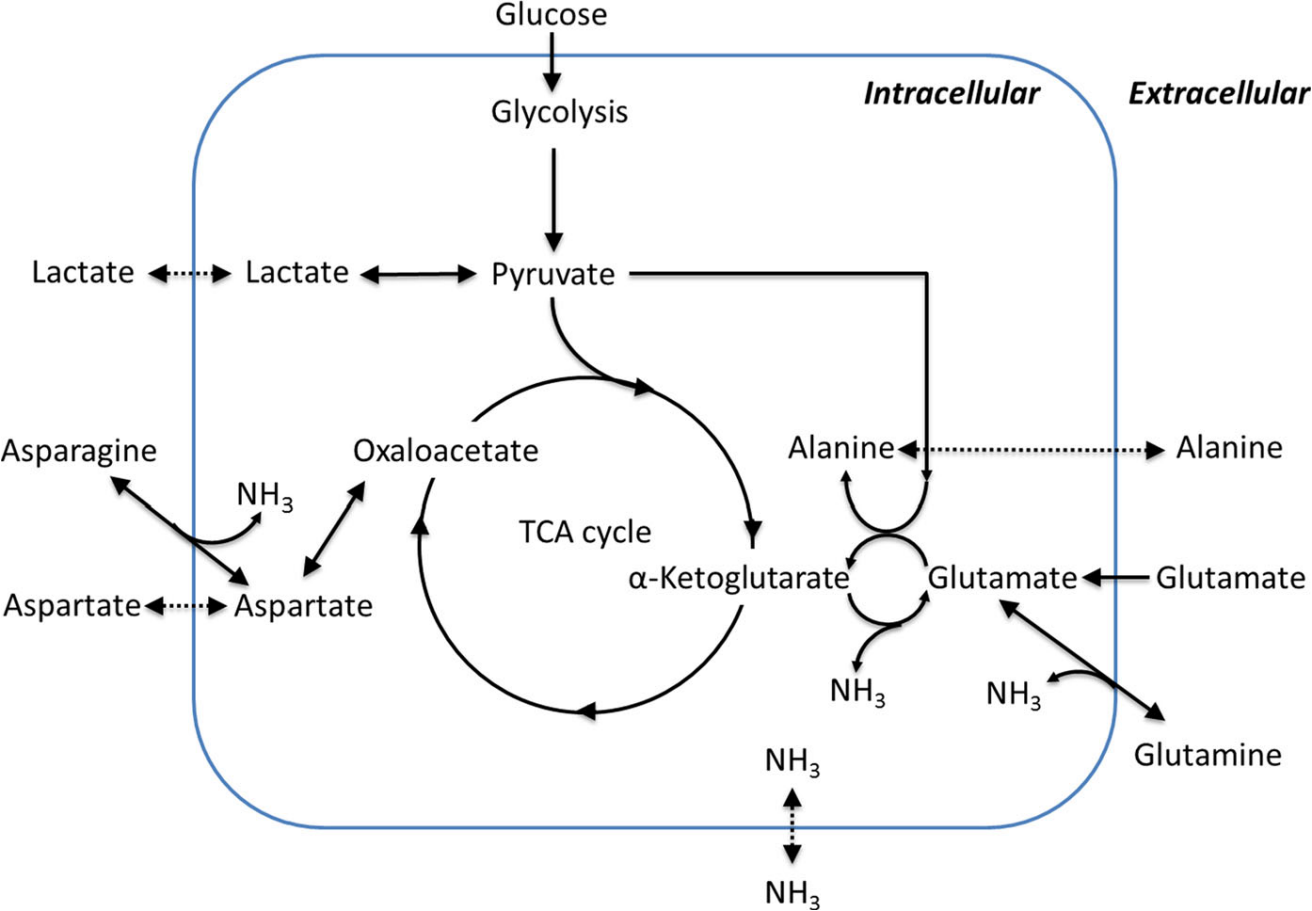
Schematic diagram of pathways related to the fates of lactate and ammonia

Correlated with the drastic increase of cell size for the BC-P clone fed by Actifeed A/B, glucose metabolic activity showed large variation for this clone (Fig. 9). Since the feeding strategy in this experiment was based on glucose consumption, the addition of other nutrients may be out of balance. In order to evaluate the balance of the feeds, we studied the relative balancing levels of the feeds for each condition **(**Fig. 10 **)**. The definition of the relative balancing level can be found in the Materials and methods “Balance of essential amino acids in feeds” section. For the growth phase of both clones, both feeds are reasonably well balanced. Only for the combination of the BC-G clone with the rich medium and feed (ActiCHO with Actifeed A/B) balancing of the feed Actifeed A/B is poorer with concentrations of essential amino acids being too high relative to the glucose concentration. Upon the transition of growth to stationary phase the specific consumption rates of amino acids changes in a different way than the specific glucose consumption rate (Fig. 8), which results in an increase of balancing level values for most of the amino acids, and explains the overfeeding for these amino acids (Fig. 6). Especially for the BC-P clone fed by Actifeed A/B feed, accumulation of essential amino acids occurred when the feed addition is based on glucose consumption, due to the fact that the specific consumption rate of glucose increased while the specific consumption rates of amino acids decreased during the stationary phase. Increased amino acid levels could result in increased catabolic breakdown, which explains the increase in ammonium levels. High ammonia and glutamic acid levels would also favor formation of alanine through the transaminase reaction (Yang and Butler 2000; Chen and Harcum 2005) and formation of glutamine through glutamine synthase, which explains the formation of these compounds in these cultures (Fig. 11).

### Optimization of fed-batch cultures

Optimization of medium and feed formulations, and feeding strategy is an important step in process development. To keep stable nutrient levels, a feed formulation that is balanced with respect to the needs of a cell line over the whole culture period is required. In this study, a glucose based feeding strategy is applied instead of following the prescribed feeding strategy by suppliers in order to obtain a fairer cross comparison between basal media and feeds. This feeding strategy resulted in overfeeding of amino acids by Actifeed A/B on both clones (Fig. 6) due to an imbalance in the feed. Notably, overfeeding of amino acids also occurred when following the protocols prescribed by the suppliers (Reinhart et al. 2015). This study demonstrates that CHO cells may display a high variation in specific glucose consumption in different basal medium-feed combinations and during different phases. The increase of the specific glucose consumption is related to the increase of cell size. Moreover, the variation of the specific glucose consumption rate does not always correlate with the specific amino acid consumption rate, in other words, they do not shift up or down at the same time and to the same extent. Thus, as was done in other studies (Ma et al. 2009; Li et al. 2012), it is better to add glucose through a separate feed from the amino acids. Part of the variation in glucose consumption rate may be caused by uncontrolled glucose levels. The high glucose levels were shown to induce inefficient use of glucose, thus result in higher glucose consumption rate and higher lactate production rate (Reinhart et al. 2015). Hence, control of glucose concentration at low values may reduce the variation and keep feeds more balanced throughout the process.

For all tested culture conditions the ratios of specific amino acids consumption rates changed upon transition from the growth phase to the stationary phase, implying that the composition of amino acids in the feed should also be changed accordingly. However, within the growth and the stationary phases, the behavior of the cells is fairly constant. Therefore, it would be possible to have two different feeds for the growth and the stationary phases. To our knowledge, a feed system design to meet different nutrient requirements for different culture phases has not been reported. Another feeding strategy would be to separate feed streams with nutrients that have the same ratio of specific uptake rates (e.g. essential amino acids, which ratios of requirement do not change much) in the same stream. Changes in specific consumption rates during, for example a phase transition, can then be addressed by adjusting the feed rates for the separate feeds. An extra challenge for feed design is the solubility of different nutrients. Using more feed streams may circumvent this problem and make the feeds more concentrated and balanced. To solve this the amino acids could also be added as di- or tripeptides. It was shown that amino acids supplemented in the form of a dipeptide or tripeptide improved their solubility, yet resulted in similar culture effects (Kishishita et al. 2015).

The outcome of clone generation at this moment remains unpredictable. Intensive screening of clones and media is therefore still the most common industrial practice. It is shown in this study that although 2 clones were generated in the same way from the same parental cell line, they can still have distinct metabolic behavior for the different medium-feed systems. Only for the essential amino acids in the growth phase, ratios of the specific consumption rates were similar for the two clones (Fig. 8a, c). Thus, for essential amino acids a single feed can be used for both clones in the growth phase.

## Conclusion

The aim of this work is to investigate the effects of different basal media and feeds on cell growth, metabolism, physiology, and mAb production for two differentially behaving clones derived from the same parental cell line. The two clones show different metabolic and physiological responses for the different medium-feed combinations that were applied. The best mAb product titer (1.2 g/L) is obtained with ActiCHO basal medium and feeds. For all the tested conditions, the specific consumption rates for amino acids and glucose are different for the growth and stationary phase. Also the ratio of specific uptake rates are different in the different phases. However, the ratios of the consumption rates for amino acids within a phase are fairly constant for each of the two clones. Cells grown in cultures fed by Actifeed A/B show an increase in cell size during the stationary phase. The size increase correlates with an increase in specific glucose consumption rate in the stationary phase, whereas for all other conditions the specific glucose consumption rate decreases in the stationary phase. The increase of specific glucose consumption rate in the stationary phase of the cultures fed by Actifeed A/B leads to overfeeding of the other nutrients, since the feeding is based on glucose. The result of this study can help to design better feed formulation and feeding strategy.

## Electronic supplementary material

Below is the link to the electronic supplementary material.

Supplementary material 1 (DOC 233 kb)

Supplementary material 2 (XLSX 70 kb)
