# Supplementary material for: Selection of chemically defined media for CHO cell fed-batch culture processes
Source: Cytotechnology. 2016 Nov 29;69(1):39–56. doi: 10.1007/s10616-016-0036-5 (PMC5264622; doi:10.1007/s10616-016-0036-5)

*Appendix 1. Representative calculation of specific consumption rates during the growth and stationary phase. The specific consumption rates are obtained from the slope by* *a plot of the total amount of a compound against the integral of viable cell numbers using linear regression. The units of the specific rates (slope values) are in fmol*cell^-1^*day^-1^. Values are taken from the histidine of BC-P clone fed-batch culture using ActiCHO + Actifeed A/B.*


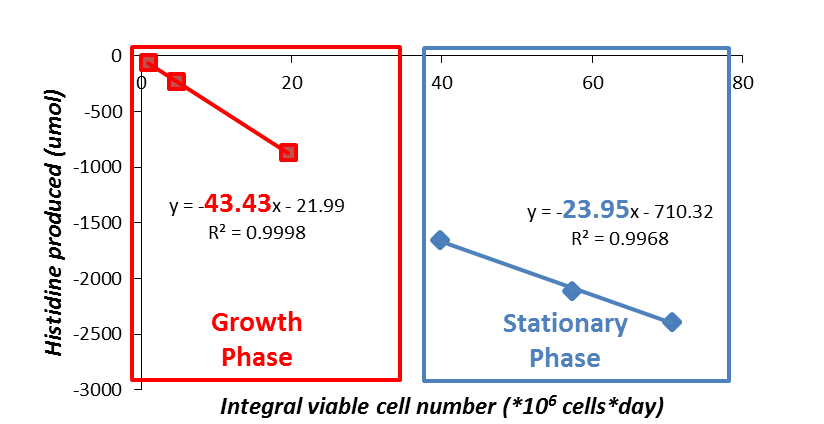


***Appendix 2.*** *Example calculation of feed supply method used in this study.*

On culture day 4, we based on data from day 3-4 to calculate the feeding for day 4-5.

From Day 3 to day4:

| **Culture day** | **Viable cell density (cell/mL)** | **Glucose concentration (mM)** |
| --- | --- | --- |
| Day 3 after feeding | 1620000 | 30.6 |
| Day 4 before feeding | 3420000 | 23.3 |

Calculate growth rate from day 3-4:


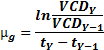


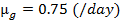


Predict the VCD for day 5 using:


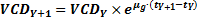


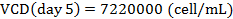


Calculate specific glucose consumption rate using:


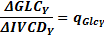


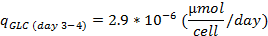


Calculate Actifeed A addition using:


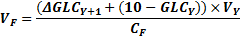


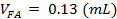


Calculate Actifeed B addition:


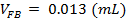


*Appendix 3. Fed-batch culture results of clone BC-G and BC-P for all possible combinations of 4 types of media and 3 feed systems. “±” indicates the deviation of the duplicate data points from the average value. Asterisks at the max viable cell density values indicate cell aggregate during the cultures. Conditions marked in blue are selected for further comparison.*


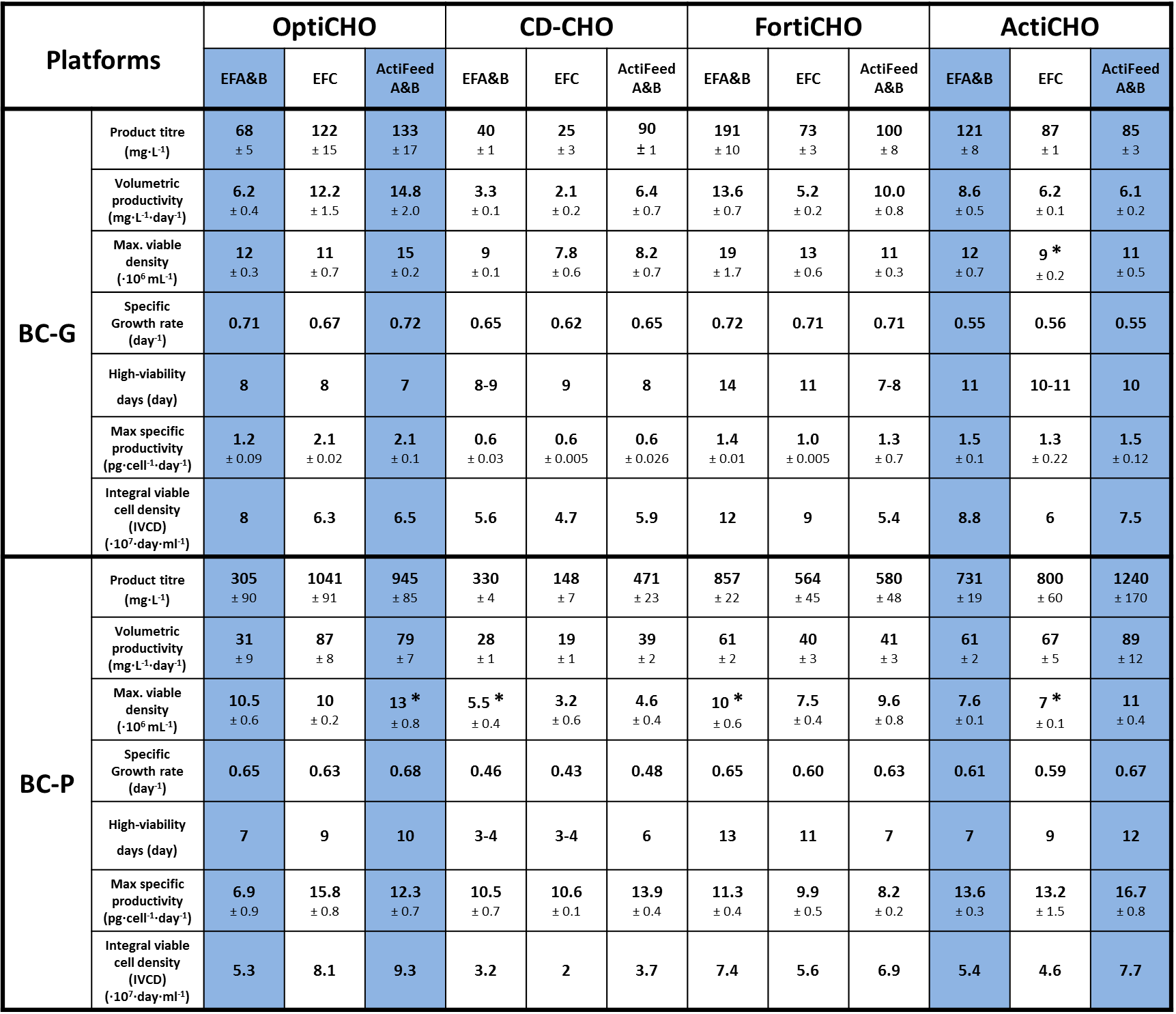

Supplement: Supplementary file 1 — Supplementary material 1 (DOC 233 kb) [file 10616_2016_36_MOESM1_ESM.doc]
